# The signature of extracellular vesicles in hypoxic breast cancer and their therapeutic engineering

**DOI:** 10.1186/s12964-024-01870-w

**Published:** 2024-10-21

**Authors:** Baiheng Zhu, Kehao Xiang, Tanghua Li, Xin Li, Fujun Shi

**Affiliations:** 1 The Second School of Clinical Medicine, Zhujiang Hospital, Southern Medical University, Guangzhou, 510280 China; 2 The First Clinical Medical School, Nanfang Hospital, Southern Medical University, Guangzhou, 510515 China; 3 Department of Breast Surgery, Zhujiang Hospital, Southern Medical University, Guangzhou, 510280 China

**Keywords:** Breast cancer, Hypoxia, Extracellular vesicles, Tumor microenvironment, Engineered EVs

## Abstract

Breast cancer (BC) currently ranks second in the global cancer incidence rate. Hypoxia is a common phenomenon in BC. Under hypoxic conditions, cells in the tumor microenvironment (TME) secrete numerous extracellular vesicles (EVs) to achieve intercellular communication and alter the metabolism of primary and metastatic tumors that shape the TME. In addition, emerging studies have indicated that hypoxia can promote resistance to tumor treatment. Engineered EVs are expected to become carriers for cancer treatment due to their high biocompatibility, low immunogenicity, high drug delivery efficiency, and ease of modification. In this review, we summarize the mechanisms of EVs in the primary TME and distant metastasis of BC under hypoxic conditions. Additionally, we highlight the potential applications of engineered EVs in mitigating the malignant phenotypes of BC cells under hypoxia.

## Key points

**•** Breast cancer, which has the second highest incidence rate of cancer in the world, has a 5-year survival rate as high as 90% without metastasis. However, once metastasis occurs, the rate decreases to 10%.
**•** Hypoxia is a significant feature of BC and impacts its TME composition, metabolic characteristics, metastasis capacity, and therapeutic resistance.
**•** Under hypoxia, the above biological processes rely mainly on the participation of EVs.
**•** Engineered EVs have significant potential to address the aforementioned clinical issues.

## Background

According to the latest global cancer report, breast cancer (BC) currently ranks as the second most common cancer worldwide and poses a serious threat to human health. Despite the availability of numerous treatment options, the mortality rate of BC remains the fourth highest among all populations and the highest among women, underscoring its significant danger [1]. As a solid tumor, BC commonly exhibits hypoxia within the tumor microenvironment (TME) during its progression. Hypoxia is closely related to carcinogenesis, tumor angiogenesis, and cellular genomic damage [2]. The transcriptional regulator hypoxia-inducible factor (HIF) is a crucial component of the cellular response to hypoxia, influencing processes such as cellular metabolism and signaling pathway expression and thereby altering cellular phenotypes and characteristics [3]. Extracellular vesicles (EVs) are nanospherical bilayer proteolipid structures that can transport various bioactive substances (including signal molecules and metabolites) between cells, facilitating intercellular communication [4, 5]. Compared with EVs from other tumors, EVs from BC have different compositions. They generally contain unique protein molecules, RNA fragments (especially microRNAs (miRNAs/miRs)), and DNA fragments, which are closely related to the progression of BC malignant phenotypes [6–8]. Under hypoxia, BC cells regulate the TME by secreting EVs, which transform the TME into an immunosuppressive environment [9]. In addition, EVs from the hypoxic BC TME facilitate cancer progression, such as by altering metabolic patterns and promoting metastasis, epithelial-to-mesenchymal transition (EMT), angiogenesis, and therapeutic resistance [10].

Many reviews have thoroughly summarized and discussed the role of EVs in the progression of tumors, including BC, as well as their diagnostic and therapeutic value [11, 12]. However, there are limited studies summarizing how the properties of EVs change under hypoxia and how these changes affect tumor development differently than under normoxic conditions. In this review, we comprehensively discuss how EVs affect BC progression under hypoxia. The various research directions for developing engineered EVs to target hypoxic BC tissue are also summarized in the last section. This review details the roles that EVs play in the mechanisms and treatments of hypoxia in BC, aiming to provide new ideas and directions for BC treatment regimens and targets.

## Hypoxia-induced EVs reshape the BC TME

**Fig. 1:**
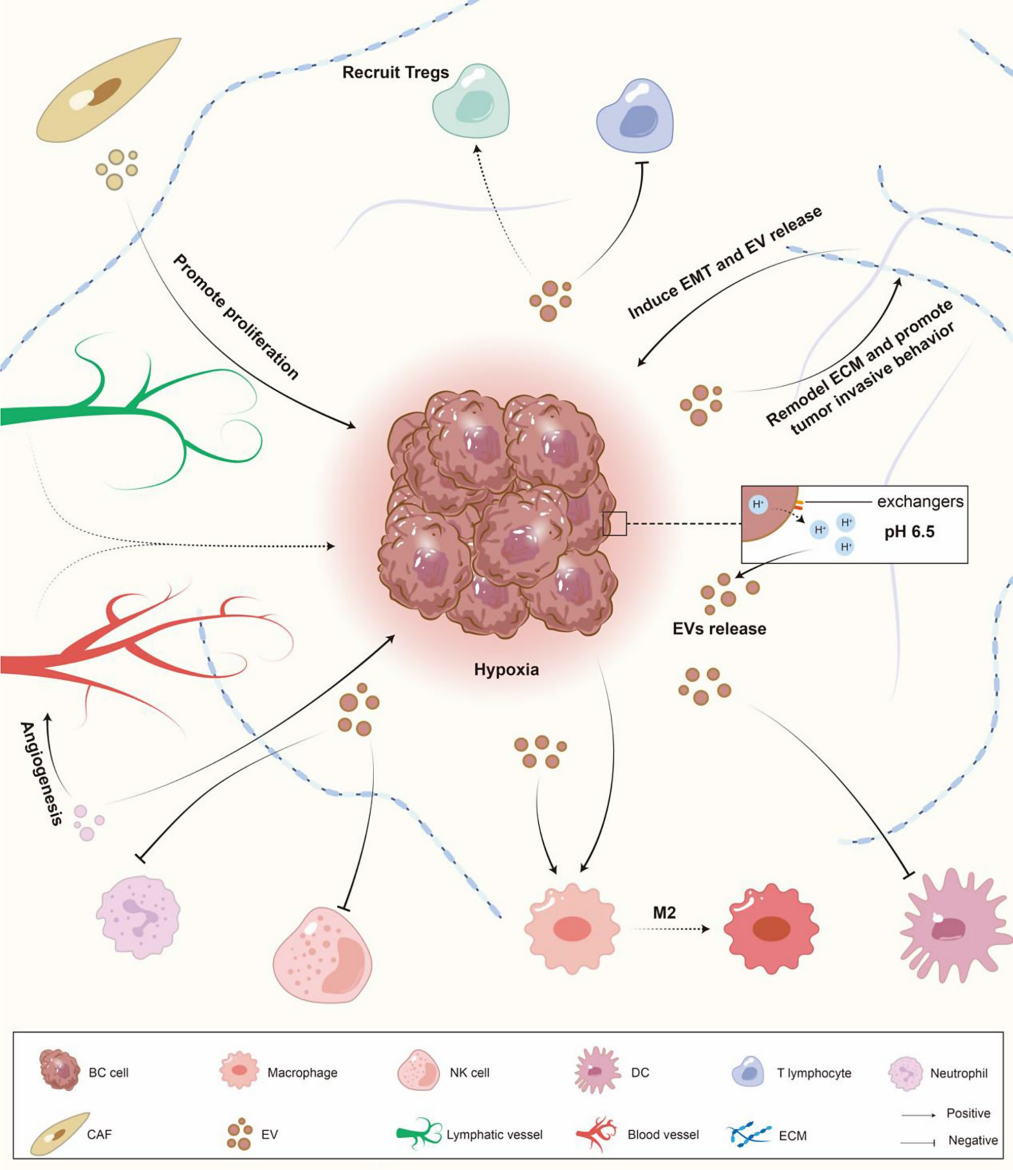
Remodeling of various components in the BC TME by hypoxic EVs. As a solid tumor, BC experiences a decrease in oxygen concentration as it grows. By altering the secretion of EVs by cell components, hypoxia induces interactions among various components in the BC TME, including BC cells, physical properties, the ECM, immune cells, fibroblasts, and lymphatic and vascular ECs, to reshape the TME. The effects of hypoxia include acidification of the TME, ECM stiffening, and inhibition of various immune cells in the TME, leading to tumor immune escape and increased metastatic capabilities of the tumor

The components of the BC TME can be divided into three parts: the physical and chemical environment, such as low oxygen and low pH; the molecular environment, including soluble extracellular signaling molecules, cytokines, and EVs; and the cellular environment [13, 14]. Immune cells, stromal cells in the matrix, and vascular cells are the three main types of cells in the BC TME [15]. Under the influence of hypoxic EVs, these components can be modulated by HIF gene expression and metabolic mode alterations [2, 16]. In this section, we summarize how the various TME components of BC react to hypoxia via EVs (Fig. 1).

### Changes in the physical and chemical properties of the TME under hypoxia

The distinct cell composition and metabolic processes in the BC TME set its physical and chemical properties apart from those of normal tissues, including hypoxia, extracellular matrix (ECM) stiffness, pH, and interstitial fluid pressure (IFP) [17]. These properties interact with each other via the mediation of EVs and can work together to form a microenvironment that is suitable for tumor growth, invasion, metastasis, angiogenesis, and drug resistance [18].

The ECM is a noncellular three-dimensional macromolecular network, and its function as a repository and binding site for bioactive molecules has been discovered recently [19]. Hypoxia can reshape the ECM by prompting cells in the TME to release EVs. For example, EVs from hypoxic triple-negative BC (TNBC) cells can facilitate promigratory morphology, invadopodia development, ECM degradation, and matrix metalloprotease (MMP) secretion to remodel the ECM and promote tumor invasion [20]. In addition to BC cells, fibroblasts in the TME can remodel the ECM. HIF-1 upregulates collagen prolyl (P4HA1 and P4HA2) and lysyl (PLOD2) hydroxylases in fibroblasts to remodel the ECM composition, alignment, and mechanical properties in response to hypoxia, making collagen fibers in the ECM thicker, denser, and more organized [21, 22]. The reshaped ECM, in turn, can also promote cancer progression by promoting EV release. In BC cells, a stiff ECM activates the Akt/Rab8 and YAP/TAZ pathways to drive EV secretion, which can promote tumor growth by activating the Notch signaling pathway and mediates proinvasive effects by engaging the MMP and focal adhesion kinase in other cancer cells [23, 24].

Similar to the ECM, there is also an interaction relationship between the TME pH and tumor cells. In accordance with the Warburg effect, hypoxia triggers the anaerobic metabolism of glucose in tumor cells, which causes extracellular acidification at approximately pH 6.5 [25, 26]. Logozzi et al. reported that acidity was a major determinant of the ability of BC cells to release EVs [27]. These EVs are related to local invasion, metastasis, angiogenesis, immune system escape, and therapeutic resistance in cancers [28].

Abnormal blood and lymphatic vessels create a hostile TME with elevated IFP, which results from high cell density, increased vascular permeability, impaired venous or lymphatic drainage, and abnormal ECM [29]. According to research by Koomullil et al., higher EV concentrations and IFP are correlated at the later stages of tumor growth [30]. However, apart from this, no further mechanisms have been found regarding the interaction between IFP and tumors via EVs.

### Hypoxic EVs reduce the antitumor ability of immune cells

In general, different molecular subtypes of BCs have different immune features. Increasing evidence suggests that EVs play a crucial role in immune infiltration in BC under hypoxic conditions [31]. This section summarizes the EV-mediated changes in immune cells in the BC TME under hypoxia.

#### Remodeling of innate immune cells

Macrophages play an indispensable role in regulating cancer immunity [32]. Emerging evidence indicates that modulators such as miRNAs in tumor-derived EVs (TD-EVs) activate various signal transduction mechanisms to aggregate and polarize macrophages [33, 34]. For example, Park et al. reported that hypoxic TD-EVs are highly rich in immunomodulatory proteins and chemokines such as colony-stimulating factor-1 (CSF-1), C–C motif chemokine ligand 2, and transforming growth factor-β (TGF-β), which can promote macrophage recruitment and M2-like polarization [35]. Specifically, for BC, circ_0001142 carried by TD-EVs and miR-142–5p, miR-183–5p, and miR-222–3p carried by endothelial cell (EC)-derived EVs are relevant to M2-like polarization, but the relationships between these factors and hypoxia need to be elucidated [36]. In fact, the impact of hypoxia on tumor cells and macrophages is bidirectional. Chen et al. reported that tumor-associated macrophages (TAMs) enhance aerobic glycolysis and apoptotic resistance in BC cells via EV transmission of a HIF-1α-stabilizing long noncoding RNA (lncRNA), thereby promoting cancer cell survival under hypoxia [37].

The natural killer (NK) cell, a member of the innate lymphoid cell family, can recognize target cells nonspecifically. Under hypoxic conditions, BC cells usually dysregulate and suppress NK cells via EVs [9]. EVs can be used by cancer cells to transport signal molecules to NK cells. For example, in hypoxic TD-EVs, researchers have shown that TGF-1 and high levels of miR-23a decrease the expression of the activating receptors NKG2D and CD107a on the NK cell surface, respectively, to inhibit NK cell antitumor function [38]. In addition to transporting signal molecules such as miRNAs, TD-EVs can affect NK cells by modulating metabolic conditions. In BC, hypoxia mediates neutral lipid accumulation in lung mesenchymal cells (MCs) via interleukin-10 (IL-10), and lipid-laden MCs transport lipids to cancer cells as well as NK cells via EVs, leading to BC lung metastasis and NK cell dysfunction. The underlying mechanism may be that glucose metabolism and oxidative phosphorylation (OXPHOS) in NK cells are impaired by lipids [39]. Lactate production is another outstanding characteristic of hypoxia metabolism [40]. TD-EVs can stimulate lactate production and secretion into the TME and lead to extracellular acidosis, which suppresses the cytotoxic function of NK cells [41]. Interestingly, in contrast to the immune inhibitory effect of hypoxic TD-EVs, hypoxic EVs from NK cells exhibit strong cytotoxicity. A recent study revealed that under hypoxia, the expression of Fas ligand, perforin, and granzyme B was increased in EVs from two NK cell lines [42]. Therefore, hypoxia can both indirectly inhibit NK cells through TD-EVs and directly promote the antitumor function of NK cells. The overall effect is worth further exploration.

As the most important type of antigen-presenting cells, dendritic cells (DCs) in the TME can be altered by hypoxia via various mechanisms [43]. In DCs, hypoxia first activates HIF-1α and then upregulates C-C chemokine receptor 5 (CCR5), IL-1, and vascular endothelial growth factor (VEGF), accumulates lactate, downregulates interferon-γ (IFN-γ) and alters the DC chemokine expression profile [44]. Through these pathways, the maturation, migration, and antigen-presenting abilities of DCs are impaired, which makes the BC TME immunosuppressive [16, 45]. However, the impact of hypoxic EVs on DCs in the TME is not yet clear, and few relevant research reports exist.

Tumor-associated neutrophils (TANs) aggregate in the TME of the primary lesion, blood, and distant metastatic organs, where they form the premetastatic niche (PMN) [15]. During hypoxia, the expression of HIF in neutrophils increases, causing them to polarize toward N2, which contributes to an immunosuppressive TME [46, 47]. Under hypoxic conditions, BC cells increase neutrophil infiltration in the TME by signaling molecules such as lncRNAs and chemokines, including the chemokine C-X-C motif ligand 1–3 (CXCL1-3), CXCL5-8, and CXCL12, and some lncRNAs have been demonstrated to be transported from BC cells to neutrophils through EVs [48, 49]. In the communication between TANs and other TME cells, EVs play an important role. N2 neutrophils release EVs containing miR-4780, which targets the SOX11 gene of other TME cells, to aggravate EMT and angiogenesis, which are believed to alleviate hypoxia [50]. TD-EVs can also modulate neutrophil early granulation to form neutrophil extracellular traps (NETs), important structures facilitating the metastasis of circulating tumor cells (CTCs) via various mechanisms [47, 51]. For example, colorectal cancer cells release EVs containing a KRAS mutation to stimulate NET formation by inducing IL-8 expression [52]. However, the EV-mediated mechanism of NETs under hypoxia has not been identified in BC.

#### Remodeling of adaptive immune cells

T lymphocytes are among the most abundant immune cell types in the BC TME [53]. The proliferation and activation of T lymphocytes can be affected by EVs in the hypoxic TME. In EVs collected from the hypoxic TME and plasma of BC patients with paclitaxel resistance, the level of heat shock protein (HSP) gp96 was significantly upregulated, which activated CD8 + T lymphocyte pyroptosis to disrupt immune surveillance [54]. Additionally, other factors from hypoxic cancer cells regulate T lymphocyte entry into the TME. BC cells can express HIF-1α to recruit regulatory T lymphocytes (Tregs) to the TME by overexpressing miR-25/93 and upregulating the levels of chemokines, such as CCR2, CCR3, CCR5, CCR7, and CCR17, under hypoxic stress [55, 56]. MiR-23, miR-125, miR-210, and Let-7a have also been shown to inhibit CD4 + and CD8 + T lymphocyte infiltration in the TME or suppress their differentiation. However, no direct EV-mediated effects on these factors have been described [57]. This suggests that relevant verification and exploration could be carried out in the future.

Current insight into the features of B lymphocytes in the BC TME is lacking. Several factors, including miR-210 and miR-125, which can impair B lymphocyte proliferation, maturation, release and antibody production, and suppress B lymphocyte-mediated immune reactions, are enriched in EVs from hypoxic cancer cells [57]. In addition, hypoxia can increase CD19 + EV release in B lymphocytes, which can damage CD8 + T lymphocytes in the TME and reduce the antitumor effect of chemotherapy [58].

#### Remodeling of fibroblasts in the TME

Cancer-associated fibroblasts (CAFs) have extensive contact with tumor cells and can affect other components in the BC TME by secreting diverse cytokines to inhibit tumor immunity, reshaping the TME structure, and promoting cancer cell proliferation as well as angiogenesis [59, 60]. Under hypoxic stress, such functions of CAFs are further enhanced in BC. CAF-derived EVs (CAF-EVs) can impact cancer cells in many ways. First, CAF-EVs modulate the protein metabolism of cancer cells. For example, under hypoxia, CAFs in the BC TME release miR-500a-5p via EVs to promote BC cell proliferation and metastasis through the targeting of ubiquitin-specific peptidase 28; thus, various cancer suppressor proteins are ubiquitinated [61, 62]. Second, CAF-EVs can transport signaling factors to regulate signaling pathways in cancer cells. Zhan et al. screened a circular RNA (circRNA) and named it circHIF1A. CircHIF1A is produced by CAFs under hypoxia and transferred into BC cells by EVs. CircHIF1A plays an important role in cancer stem cell (CSC) properties, sponging miR-580-5p by regulating CD44 expression [63]. Finally, CAFs can transport modulators via EVs to alter BC cells at the genetic level. Liu et al. reported that CAF-EVs containing miR-7641 promoted BC stemness and glycolysis by targeting HIF-1α under hypoxia [64]. Additionally, autophagy-associated adhesion G protein-coupled receptor G2 (GPR64) is enriched in hypoxic CAF-EVs, which stimulate noncanonical NF-κB signaling to upregulate the expression of MMP-9 and IL-8 in recipient BC cells, enhancing the invasive ability of BC cells [65].

In this section, we reviewed the roles that various components in the BC TME play and the mechanisms by which EVs regulate each other under hypoxia. However, the roles of B lymphocytes and neutrophils remain largely unclear. In the future, in-depth research on the interaction mechanisms between TME components under hypoxia will be beneficial for identifying new valuable targets for the treatment of BC.

## Hypoxia-induced EVs participate in BC metabolism

As BC tumors grow, hypoxia becomes a significant characteristic of the TME. Compared with normoxic BC cells, BC cells under hypoxic conditions exhibit metabolic reprogramming, which is characterized primarily by the involvement of HIF in glycolysis and the induction of other factors under hypoxic conditions [66, 67]. Here, we summarized the alteration in hypoxic glucose metabolism, amino acid metabolism, and lipid metabolism in BC cells, in which EVs play vital roles.

### Alterations in glycolysis caused by hypoxia and EVs

**Fig. 2:**
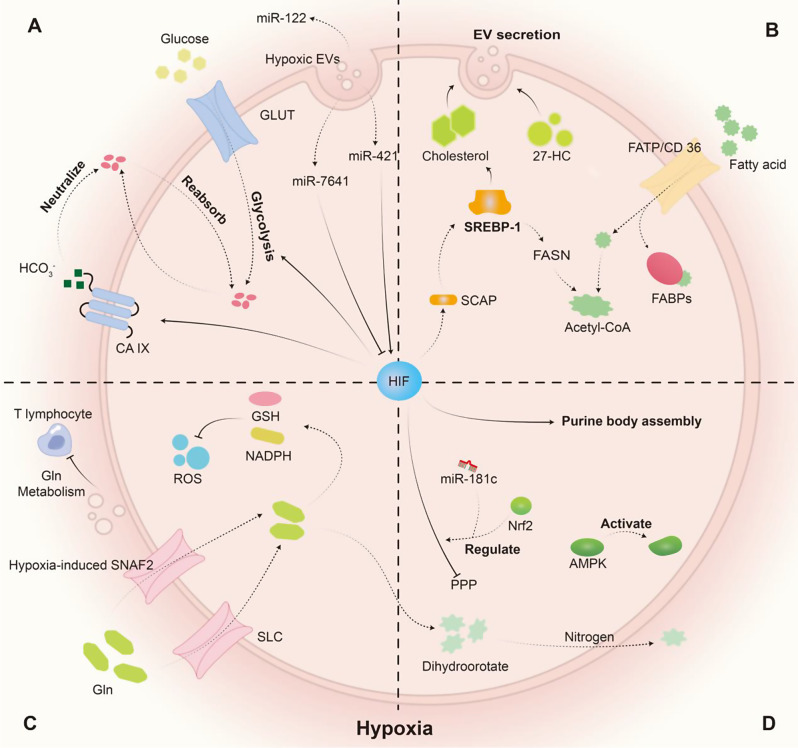
The impact of hypoxia-induced EVs on the metabolism of various substances in BC cells. (**A**) The effects of hypoxia and EVs on glucose metabolism. HIF expression affects the number of transporters such as GLUT and CA IX, promoting glycolysis and increasing lactate production in BC cells. EVs and their contents regulate glucose metabolism. (**B**) The effects of hypoxia and EVs on lipid metabolism. FATP/CD36 and FABPs are the main sites of action. (**C**) The effects of hypoxia and EVs on protein metabolism. Gln is the main protein affected. Membrane surface receptors such as SNAF2 and SLC are easily regulated by hypoxia and thus affect gln metabolism. Hypoxia increases BC cell uptake of gln. (**D**) The effects of hypoxia on nucleotide metabolism in BC cells. HIF is the main influencing factor, which downregulates the synthesis of purines and pyrimidines by affecting the PPP, AMPK, and assembly of purine bodies

Under aerobic conditions, BC tends to rely on faster energy acquisition through glycolysis rather than OXPHOS, a phenomenon known as the Warburg effect [68]. Under normoxic conditions, myeloid immune cells tend to uptake more glucose than tumor cells, while tumor cells preferentially take up glutamine (Gln), which is a phenomenon observed in the TME of various cancers [69]. However, hypoxia leads to the upregulation of glucose transporters (GLUTs), which consequently take up more glucose in cancer cells [70]. In the process of glucose uptake from the extracellular space to lactate formation, HIF is involved in regulating the activity of certain enzymes under hypoxic conditions, including GLUT, pyruvate kinase M (PKM), and lactate dehydrogenase-A (LDH-A) [71, 72]. Under hypoxia, BC cells secrete lactate and EVs carrying miR-122, which is a relatively unique EV of BC, into the TME, leading to acidosis and inhibiting the uptake of glucose by surrounding tissues to create an immunosuppressive environment and facilitate metastasis [8, 73, 74]. Tumor cells can upregulate carbonic anhydrase 9 (CA IX) activity through HIF to neutralize lactate [75]. The expression of HIF-1α can be regulated by different EVs originating from CAFs. CAF-derived miR-7641-enriched EVs have been shown to directly inhibit the expression of HIF-1α, whereas CAF-derived miR-421-enriched EVs upregulate HIF-1α expression [76–78]. Recent studies have shown that EVs can alter glycolysis in various tumor cells by targeting HIF-1α, including EVs with ZFPM2-AS1 in liver cancer, EVs with miR-29a-3p in glioma, and EVs with circSHKBP1 in non-small cell lung cancer [79–81]. Whether similar mechanisms exist in BC remains to be studied (Fig. 2A).

### Alterations in lipid metabolism caused by hypoxia and EVs

Hypoxic conditions can be involved in almost all aspects of lipid metabolism in BC [82, 83]. Since the generation of EVs primarily involves changes in the composition of the plasma membrane and endoplasmic reticulum membrane, factors influencing the generation of the cellular membrane may also impact the generation of EVs, which mainly involve components such as sphingomyelin, phosphatidylserine, and cholesterol in EVs [84, 85].

Lipid metabolism starts with the uptake of fatty acids. This process is carried out with the assistance of the fatty acid transport protein (FATP)-mediated pathway and the fatty acid translocase/CD 36-mediated pathway, as well as fatty acid binding proteins (FABPs) [86, 87]. In BC, fatty acid synthetase (FASN) is a key metabolic enzyme involved in fatty acid synthesis (FAS), with a positive impact on the proliferation, migration, and invasion of BC [88, 89]. Mechanically, when FASN is downregulated, its substrate acetyl-CoA accumulates, leading to the death of hypoxic cells [90]. Under hypoxic conditions, the HIF-1/SCAP/sterol regulatory element-binding protein-1 (SREBP-1) pathway is activated, leading to the upregulation of FASN expression, which increases cellular lipid metabolism to promote BC cell proliferation and provides lipid raw materials for EV formation [91, 92]. Conversely, EVs can regulate FASN. Li et al. reported that EVs containing miR-10b-5p can significantly increase FASN expression by activating the PI3K/Akt pathway in tumor cells [93]. In addition to FASN, hypoxia-induced EVs affect other lipid metabolism-related enzymes. Hypoxic adipocyte-derived EVs transfer low levels of miR-433-3p expression to tumor cells, which targets stearoyl-CoA desaturase 1 to facilitate tumor proliferation, migration, and lipid accumulation [93].

Cholesterol is a fundamental structural component of the cell and EV membrane, influencing their permeability, fluidity, and phase behavior [85, 94]. Hypoxia is believed to regulate cholesterol by increasing HIF-1α generation, which induces the upregulation of SREBP-1, leading to cholesterol alteration in cells [95, 96]. Further research has demonstrated that 27-hydroxycholesterol (27-HC), a cholesterol metabolite, potentially affects BC progression by increasing EV secretion [97]. Furthermore, EVs secreted by neutrophils treated with 27-HC have been shown to promote the development of BC, revealing the potential value of 27-HC in BC [98] (Fig. 2B).

### Alterations in amino acid metabolism caused by hypoxia and EVs

Amino acids are essential nutrients for protein synthesis and are required for cancer cell proliferation [99]. Under hypoxic conditions, there is a significant increase in the concentration of amino acids within cancer cells, while amino acids in EVs have the potential to serve as prognostic tumor markers [100–102]. Therefore, we summarize the impact of hypoxia and EVs on amino acid metabolism in BC, in which Gln plays a pivotal role.

Glutaminolysis plays an increasingly significant role in the progression of BC because Gln can activate cytotoxic lymphocytes (CTLs) and differentiate CD4 + T lymphocytes into inflammatory subtypes [103]. Gln is primarily obtained through exogenous uptake [104]. Hypoxia-induced upregulation of SNAF2 (or SLC38A2) expression via HIF-1α increases Gln uptake, resulting in complete resistance of BC cells to estrogen and certain VEGF therapies [105]. In addition, Gln serves as a substrate for the synthesis of glutathione (GSH) and NADPH, which help cancer cells counteract reactive oxygen species (ROS) accumulation induced by hypoxia and increase cellular autophagy [106–109]. There is evidence suggesting that EVs participate in the regulation of Gln metabolism. In addition, BC cells can utilize EVs containing miR-105 as a means of self-regulation through communication with CAFs, resulting in increased Gln metabolism to acquire better adaptation to the hypoxic environment [110]. Furthermore, hypoxic tumor cells can increase mTOR and adenosine 5’-monophosphate-activated protein kinase (AMPK) α activation in target cells through EVs, promoting amino acid synthesis and uptake to increase protein synthesis [111]. (Fig. 2C)

### Alterations in Nucleotide metabolism caused by hypoxia

Another significant metabolic reprogramming feature of cancer cells is increased nucleotide metabolism to meet their increased proliferation demands [112]. The products generated from the reprogramming of nucleotide metabolism, including miRNAs, circRNAs, and lncRNAs, can be utilized for intracellular metabolic reprogramming as well as transferred to neighboring and distant cells via EVs [113]. Hypoxia generally inhibits nucleotide metabolism. In this section, we summarize the regulation of nucleotide metabolism by hypoxia and EVs.

Hypoxia does not increase de novo purine synthesis. Hypoxic BC cells can regulate HIF-1α in surrounding cells by secreting EVs with miR-181c and nuclear factor erythroid 2-related factor 2 (Nrf2) to reduce 5-phosphoribose synthesis by inhibiting the pentose phosphate pathway (PPP) [100, 114, 115]. HIF-1 can promote the assembly of purine bodies, the active centers for purine synthesis, but subsequent purine synthesis does not significantly increase [116, 117].

Pyrimidine biosynthesis requires Gln and is regulated by the pyrimidinosome. AMPK is a major regulatory factor of the pyrimidinosome and can be activated under hypoxic conditions, indicating that hypoxia may be an indirect regulatory factor of the pyrimidinosome [118, 119]. During hypoxia, dihydroorotate carries nitrogen from Gln and is excreted extracellularly to maintain the carbon-nitrogen balance of intracellular Gln [120]. This phenomenon downregulates de novo pyrimidine synthesis (Fig. 2D).

In general, hypoxia remodels the metabolic characteristics of BC, and EVs play important roles in regulating specific processes. When the environment of a tumor is no longer suitable for growth, it tends to undergo a series of metabolic changes to adapt to the environment and prepare for metastasis.

### Metabolic reprogramming of metastasis

Further tumor growth exacerbates hypoxia and nutrient deficiency at the primary tumor site, forcing some cancer cells to undergo metastasis to alleviate this pressure [121]. Only when a metastatic organ forms a microenvironment suitable for tumor cell growth can CTCs settle and generate metastatic foci. The formation of the microenvironment before tumor metastasis mainly involves EVs [122]. Metabolic changes in BC metastasis occur during PMN formation and metabolic adaptation to relevantly rich oxygen after metastasis [123].

#### PMN formation

Before lung metastasis in BC, macrophages and neutrophils accumulate in the lung through the nonclassical WNT/planar PCP-RACI-JNK axis, forming a tumor-suppressive microenvironment [123]. TD-EVs promote increased glucose uptake in macrophages through toll-like receptor 2 and NF-κB signaling pathway activation, whereas the promotion of glycolysis induced by nitric oxide synthase 2 affects the expression of an immunosuppressive phenotype in macrophages [124]. Moreover, BC cell-derived LC3 + EVs can recruit monocytes and suppress T lymphocyte function in the lung PMN [125]. In addition to immune cells, the expression of COX-2 in lung fibroblasts is involved in PMN formation in BC lung metastasis [126].

In BC bone metastasis, the main effector cells involved in PMN formation are osteoblasts and osteoclasts. Both the classical WNT signaling pathway in osteoblasts and the enhancement of osteoclast activity mediated by BC cell-derived EV miR-21 through the PDCD4 protein contribute to PMN formation in BC bone metastasis [123, 127].

The primary cells involved in PMN remodeling in BC brain metastasis are microglial cells and astrocytes. M2 microglial cells can promote the invasion and brain colonization of metastatic BC [128]. BC-derived EVs with high levels of miR-122 can downregulate glycolysis in cells such as neurons and astrocytes by targeting PKM and GLUT1, and then accomplish PMN formation by reprogramming the energy metabolism of nontumor cells and providing tumor cells with more glucose and energy for invasiveness [8].

In BC liver metastasis, TD-EVs play a significant role in the PMN. BC-derived EVs can activate liver sinusoidal ECs, disrupting vascular barriers [129]. This remodeling contributes to the formation of the PMN. Furthermore, NET-DNA induces the migration, adhesion, and proliferation of tumor cells toward the liver by interacting with CCDC25 on BC cells [130].

The cells involved primarily in the BC lymph node PMN include B lymphocytes and macrophages. The primary tumor leads to the accumulation of B lymphocytes in the draining lymph node, and these B lymphocytes selectively promote lymph node metastasis by producing pathogenic IgG that targets the glycosylated membrane protein HSPA4, which eventually activates CXCR4/SDF1α axis-induced metastasis [131]. TD-EVs can increase glycolysis in lymph node macrophages, leading to increased programmed cell death ligand 1 (PD-L1) expression and the development of an immunosuppressive phenotype [124].

#### Metabolic alterations in CTCs

When normal breast epithelial cells detach from the adhesive protein-rich basement membrane and enter the circulation, they undergo the inhibition of normal cell survival signals and changes in the cytoskeleton, which is known as anoikis [132]. When BC cells are in a hypoxic state and enter the circulation, they must resist oxidative stress to survive. After entering the circulation, with respect to intracellular antioxidation, the overexpression of HER2 and high levels of lactate within CTCs can drive the upregulation of the PPP, which leads to increases in the levels of NADPH and GSH [133, 134]. Conversely, upon inhibition of HER2 by drugs, the intracellular levels of NADPH decrease, leading to the accumulation of intracellular ROS [135]. The robust antioxidation system of TNBC can rapidly synthesize a large amount of GSH to counteract the transient increase in ROS and the enzymes involved, including malic enzyme 1 and nicotinamide phosphoribosyltransferase, which do not depend on HER2 [136]. Extracellular antioxidation in CTCs relies mainly on the action of pyruvate, and high levels of pyruvate and lactate induce HIF-1α, thereby maintaining the activity of CTCs and cell clusters [134]. Early tumor hypoxia may induce the formation of CTC clusters with high metastatic potential through the HIF-1α pathway [137]. Interestingly, EVs derived from BC containing miR-122 inhibit glucose uptake in nontumor cells, which may provide CTCs with more opportunities to undergo metabolic reprogramming under hypoxic conditions [8].

Once CTCs have successfully established themselves in a metastatic site, they undergo metabolic reprogramming to adapt to the new microenvironment. Common metastatic sites of BC include the brain, liver, lung, bone, and lymph nodes. In general, after CTCs reach metastatic organs, their metabolic pattern changes. Owing to the normoxia of metastatic organs in the early stage, BC cells tend to upregulate the level of OXPHOS to meet the energy requirements for metastatic growth more efficiently [123, 138]. However, in liver metastasis of BC, in the presence of EVs containing miR-335, BC inhibits the tricarboxylic acid cycle and OXPHOS while increasing the level of glycolysis [139–141].

## Hypoxia-induced EVs facilitate invasion and metastasis in BC

**Fig. 3:**
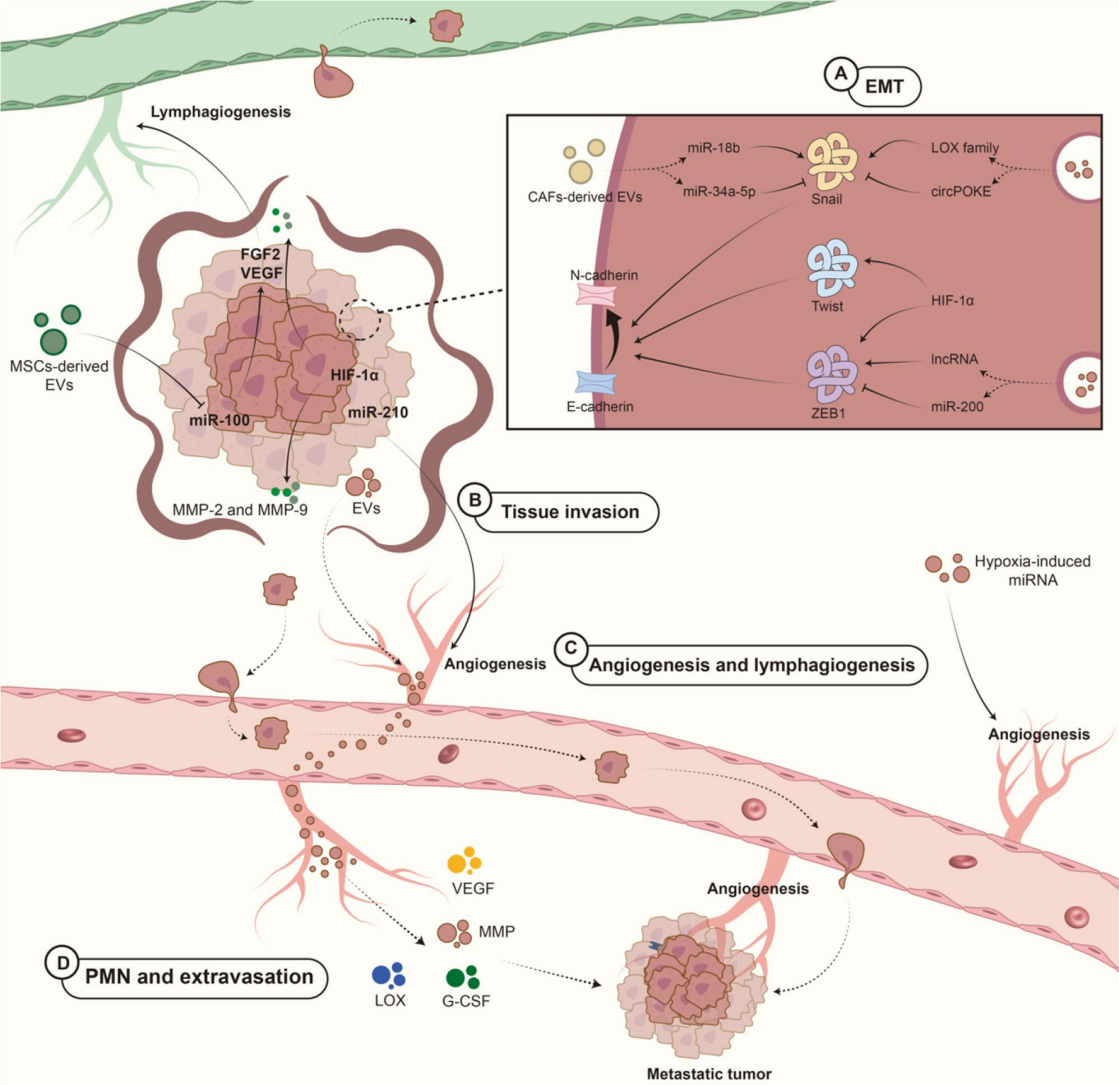
The collaborative effects of hypoxia and EVs promote BC metastasis. (**A**) Activation of endogenous factors such as HIF and EVs from CAFs can induce EMT in BC cells. (**B**) MMPs can shed BC cells from the BM of the original tissue and prepare them for subsequent distant metastasis. (**C**) BC can promote lymphangiogenesis and angiogenesis, and hypoxia-associated EVs can regulate both processes. The main mode of distant metastasis of BC is through lymphatic vessels. (**D**) Once the tumor reaches the metastatic site, a series of changes occur to facilitate better growth in the new environment. Hypoxia upregulates the levels of VEGF, G-CSF, LOX, and MMP, which together form a complex network of interactions and create conditions for BC development during metastasis

Invasion and metastasis are the leading causes of BC mortality. Up to 30% of BC patients progress to metastasis after diagnosis and primary treatments and 90% of all deaths in BC patients are related to metastasis [142]. There are three routes of metastasis in BC: direct invasion, lymph node metastasis, and hematogenous metastasis [143]. Hypoxia can increase the invasiveness and metastasis ability of BC [144]. Moreover, hypoxia induces the release of EVs in BC, which contain HIF-1α target genes [145]. Additionally, these EVs present elevated levels of several HIF-1α-inducible proteins [146]. These studies indicate the potential roles of EVs under hypoxic conditions. Here, we summarized the roles that hypoxia-induced EVs play in the continuous process of BC metastasis to target organs (Fig. 3).

### Effects of hypoxia and EVs on EMT

The whole “journey” of cancer metastasis starts with EMT [147]. The main process of EMT is the transition from E-cadherin to N-cadherin, enabling the polarity of the cell to be exchanged for aggressiveness; however, the loss of E-cadherin is not necessary for the BC EMT [148, 149]. Cancer EMT involves the activation of various transcription factors and changes in the expression of specific miRNAs and lncRNAs in hypoxia-induced EVs [146]. Hypoxia-induced EVs mainly participate in the SMAD pathway, resulting in a change in the expression of E-cadherin, and this process can be modulated by many factors involving TWIST, Snail, and zinc-finger E-box binding 1 (ZEB1) as well as ZEB2 [150]. The abnormal reactivation of EMT is linked to the malignant characteristics of tumor cells [151].

Hypoxia and EVs are both involved in the regulation of ZEB. Evidence has shown that ZEB1 expression is influenced by lncRNAs and the miR-200 family. Yuan et al. reported that EV-derived lncRNAs, such as ZFAS1, can positively regulate ZEB1 and ZEB2 under hypoxia, inducing EMT and promoting invasion in cancer metastases [152, 153]. The function of the miR-200 family is to regulate the SMAD pathway both directly and indirectly [154]. Under hypoxic conditions, miR-200b-3p induces angiogenesis to promote tumor metastasis [155].

Both Snail and TWIST are important regulators of EMT. As HIF-1α induces Snail expression both indirectly and directly, Snail can be a downstream target of modulators, including histone deacetylase 3, hypoxia response element, and the lysyl oxidase (LOX) family [156–158]. While the LOX family promotes Snail stabilization, EV-derived circPOKE, a BC circRNA, downregulates Snail stabilization, thus decreasing BC cell EMT [157, 159].

### Local tissue invasion and intravasation alterations

After EMT confers an aggressive phenotype on tumor cells, tumor cells then complete metastasis by invading surrounding tissues and attracting vessels. ECM remodeling can be regulated by hypoxia-induced EVs [160]. The basement membrane, an important component of the ECM, stands between cancer tissues and circulation. However, the barrier can be torn by cancer-derived MMPs, which mainly include MMP-2 and MMP-9, in BC [161]. Studies have shown that HIF-1 can upregulate or activate MMP-2 and MMP-9 [162, 163]. EVs, which mediate intercellular communication, also participate in this process. Hypoxic BC-derived EVs and CAF-EVs have been shown to upregulate MMP-2 and MMP-9 in BC cells, respectively [164, 165].

In addition to invading tissues, tumor cells need to build “escape routes”, which include angiogenesis and lymphangiogenesis, to complete metastasis. Many studies have suggested that hypoxia-induced miRNAs in EVs are involved in the regulation of BC cell angiogenesis. Hypoxia-induced EV-derived miR-210, produced by HIF-1α stimulation, can promote angiogenesis in BC, which can be regulated by neutral sphingomyelinase and tissue inhibitor of metalloproteinases-1 [166–168]. Compared with blood vessel metastasis, lymphatic metastasis accounts for a greater proportion of BC cases, and this process involves HIF-1α, VEGF-A, and VEGF-D under hypoxic conditions [169]. Because lymphatic vessels are typically distant from oxygen-carrying blood vessels, the lymphatic system itself is naturally exposed to a hypoxic environment [170]. When hypoxia occurs, ECs express more lymphangiogenesis factors, such as fibroblast growth factor 2 (FGF 2) and VEGF-C, and promote lymphatic dissemination [170]. In both approaches, FGF and VEGF are upregulated in ECs through contact with hypoxia-treated adipose-derived mesenchymal stem cell (ADSC)-derived EVs, and increased expression of miR-31 and let-7 in hypoxia-treated ADSC-EVs can activate the protein kinase A system signaling pathway, resulting in increased expression of VEGF and VEGF receptors [171–173]. Another study revealed that mesenchymal stem cell (MSC)-derived EVs rich in miR-100 downregulate VEGF produced by the mTOR/HIF-1α/VEGF signaling axis [174].

### Alterations in cancer cell PMN formation and extravasation

After a tumor cell enters the vessel, it needs to find a suitable place to resettle, which is called extravasation. Notably, tumor cells must remodel the metastatic location to better adjust to the environment before extravasation, which is PMN formation. As previously mentioned, the formation of BC PMNs in different target organs occurs through partially distinct mechanisms. Notably, hypoxia triggers the upregulation of many factors, such as HIFs, VEGFs, granulocyte-CSF (G-CSF), LOX, and MMPs, which have the potential to initiate and control the formation of the PMN [10, 175].

The final stage of metastasis is extravasation. In this process, cancer cells must adhere to ECs and pass through the EC-EC interactions with the assistance of HIF-1α [176]. Angiopoietin-like 4 (ANGPTL4), which is induced by hypoxia in a HIF-1-dependent manner, disrupts vascular EC-EC junctions and facilitates the extravasation of cancer cells [176, 177]. Recent studies have suggested that the high expression of ANGPTL4 in BC indicates a greater risk of lung, brain, and liver metastasis, suggesting a poor prognosis in BC patients [178, 179].

### The application prospects of EVs in BC metastasis diagnosis

Compared with EVs from other tumors, EVs from metastatic BC often have distinct expression levels of biomarkers such as HER2, Annexin A2, ANGPT1, and insulin-like growth factor receptor, which aid in BC liquid biopsy; however, these biomarkers cannot fully encompass the cargo of hypoxia-induced EVs [7, 180]. According to the above process, hypoxia-induced EVs play critical roles in BC metastasis, which suggests their potential diagnostic value. Thus, we concluded that hypoxia-induced EVs can serve as diagnostic biomarkers. MiR-20b, which is present in plasma-derived EVs, is strongly related to VEGF expression via HIF-1α and signal transducer/activator of transcription (STAT) and is significantly different from cell-free miRNAs [180–182]. In addition, miR-373 in circulating EVs is a promising diagnostic marker that can facilitate TNBC diagnosis and activate the TXNIP/HIF-1α/TWIST signaling axis to drive BC EMT [183, 184]. These circulating EV contents facilitate tumor angiogenesis and EMT and promote BC metastasis while adapting to hypoxia. Therefore, the value of these circulating EVs for the early diagnosis of BC metastasis is worth further exploration.

In fact, according to ClinicalTrial.gov (https://clinicaltrials.gov/), clinical trials have been conducted to explore the use of EVs to assist in the diagnosis and prediction of BC prognosis, which is also the primary direction of ongoing clinical trials involving EVs in BC. There are currently 11 related ongoing clinical trials, most of which aim to identify EVs with early diagnostic significance for the occurrence and metastasis of BC. Another focus of the trials is to screen out EVs with predictive value for the efficacy of treatments such as neoadjuvant chemotherapy and the prognosis of BC patients. Currently, most of the trials are still in the recruitment stage, with intervention studies accounting for a slightly greater proportion of the research types (Table 1). The abovementioned basic mechanism studies and ongoing clinical trials both suggest that the use of EVs to assist in the diagnosis and prediction of BC prognosis has notable clinical significance and is worthy of further in-depth study and translation to application.

**Table 1:** Ongoing clinical trials involving EVs in BC

NCT number	Official title	Study type	Current status
NCT04258735	Genetic characteristics of metastatic breast cancer patients	Interventional	Unknown
NCT05286684	Feasibility of exosome analysis in cerebrospinal fluid during the diagnostic workup of metastatic meningitis from breast cancer	Interventional	Recruiting
NCT04530890	Interest of circulating tumor DNA in digestive and gynecologic/breast cancer	Interventional	Recruiting
NCT02977468	Effects of MK-3475 (Pembrolizumab) on the breast tumor microenvironment in triple negative breast cancer with and without intra-operative RT: a window of opportunity study	Interventional	Recruiting
NCT05955521	Development of a prognostic and predictive biomarker for locally advanced breast cancer patients treated with neoadjuvant chemotherapy using exosome	Interventional	Active, not recruiting
NCT04781062	Development of a horizontal data integration classifier for noninvasive early diagnosis of breast cancer	Interventional	Active, not recruiting
NCT05453604	Evaluation protocols for isolation of analytes from urine for future oncology applications	Interventional	Active, not recruiting
NCT05831397	Extracellular vesicles as a diagnostic and prognostic biomarker of neoadjuvant chemotherapy (NAC) in breast cancer patients	Observational	Recruiting
NCT05798338	Characterization of extracellular vesicles in breast cancer patients’ plasma by single molecule detection array (SiMoA) digital ELISA	Observational	Recruiting
NCT05417048	MiRNAs of circulating glycosylated extracellular vesicles as biomarkers for early diagnosis of breast cancer patients	Observational	Recruiting
NCT04288141	HERdi PREDICT: a pilot study to measure the expression of the HER2-HER3 dimer in samples from patients with HER2 positive breast cancer receiving HER2 targeted therapies	Observational	Recruiting

## Hypoxia-induced EVs contribute to therapeutic resistance

Currently, a variety of treatments for BC have been used clinically, including surgery, chemotherapy, and radiotherapy, as well as personalized treatments for certain BC subtypes, such as endocrine therapy for luminal A and luminal B BC and anti-HER2 therapy for HER2 + BC [185]. However, all treatments might face the dilemma of therapeutic resistance after a few courses, which is becoming the dominant limiting factor in obtaining a clinical cure [186]. By remodeling the TME and reprogramming metabolism, hypoxia and EVs play important roles in the formation and spread of drug resistance in BC cells [187, 188]. In this section, these relevant mechanisms are explained and summarized.

### Hypoxic EVs mediate chemotherapy resistance

**Fig. 4:**
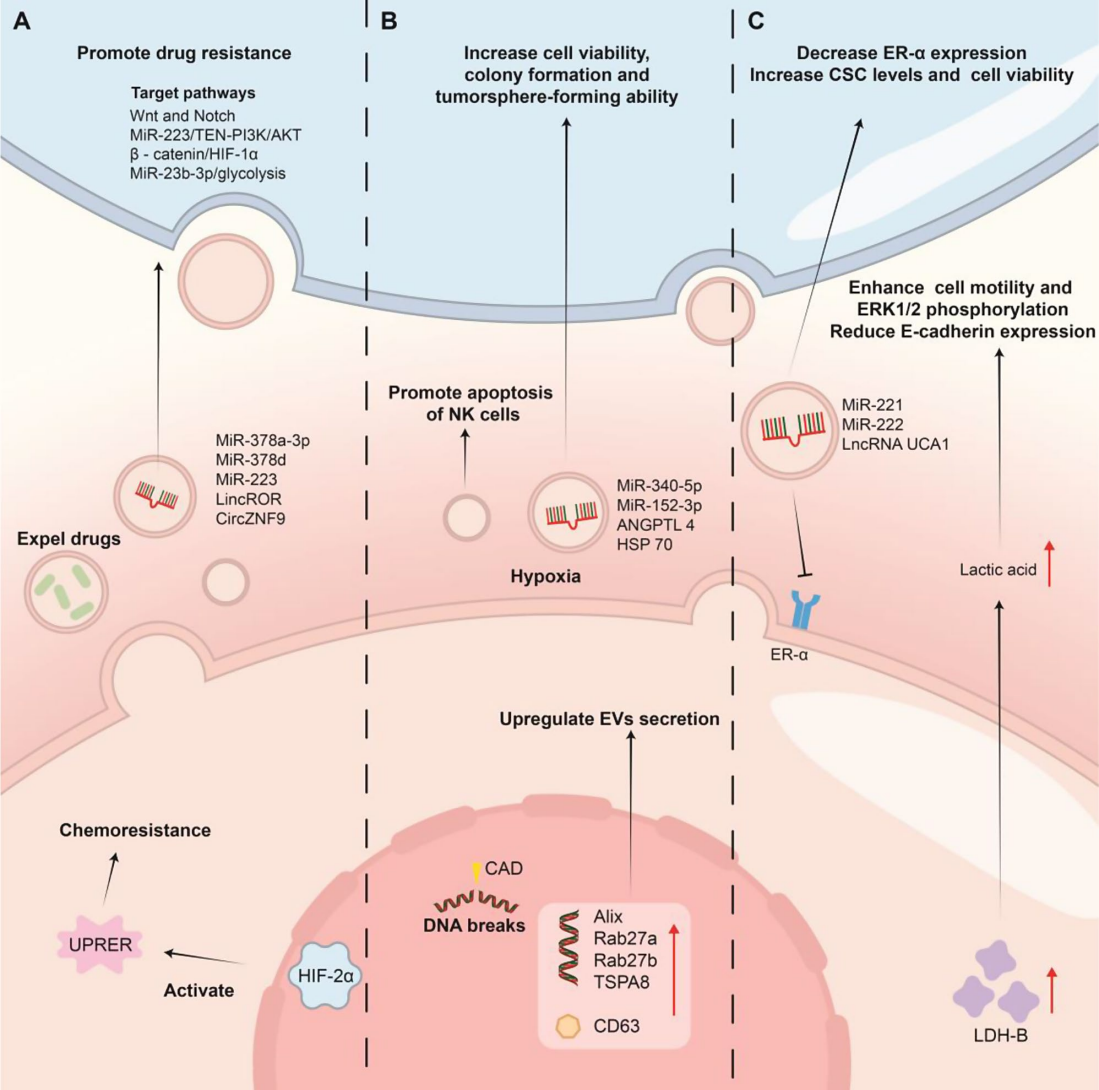
The impact of hypoxia and EVs on the therapeutic resistance of BC. (**A**) HIF-2α is a key component in hypoxia-induced chemoresistance in BC; their activation also leads to the secretion of relevant EVs to increase resistance. (**B**) Radiotherapy primarily disrupts BC DNA. Radiation stimulates the upregulation of genes related to EV secretion, promoting the release of EVs to generate resistance. (**C**) Increased lactate-related metabolism and the release of specific EVs under hypoxia induce BC to downregulate hormone receptor expression levels and increase CSC levels, resulting in resistance to endocrine therapy

Cancer cells can develop resistance under chemotherapy pressure. Hypoxia affects BC drug resistance through mechanisms such as modulating tumor metabolism, mediating the activity of different signaling pathways, and controlling drug efflux [189] (Fig. 4A). First, hypoxia-associated TD-EVs regulate the metabolism of surrounding tumor cells, especially by promoting glycolysis and FAS, which lead to alterations in chemoresistance. However, such a phenomenon has only been discovered in ovarian cancer, and evidence that EVs mediate the ability of BC to acquire chemoresistance by regulating metabolism is lacking [190]. Second, hypoxia can alter the expression of different signaling pathways in tumor cells to induce drug resistance, and EVs from these cells can deliver signaling molecules to expand the range of such effects. HIF is one of the upstream targets of these pathways. An increase in the HIF-2α-related endoplasmic reticulum unfolded protein response and HIF-1α-induced antiapoptotic protein expression are mechanisms that enable tumors, including BC, to acquire resistance to Doxorubicin [191, 192]. In addition, the signaling molecules encapsulated by hypoxia-induced EVs related to the HIF pathway include lincROR and circZNF9, which respectively activate the β-catenin/HIF-1α positive feedback loop and bind to miR-23b-3p to increase glycolysis, endowing target cells with chemotherapy resistance [193, 194]. In addition to HIF, hypoxia-induced EVs can transport different molecules to activate other signaling pathways within target cells to transmit chemotherapy resistance, including miR223, which activates the miR-223/TEN-PI3K/AKT signaling pathway, as well as miR-378a-3p and miR-378d, which are induced by the zeste homolog 2/STAT3 axis, to activate the WNT and Notch stem cell pathways [195, 196]. Third, hypoxia and EVs can enhance the ability of tumor cells to expel drugs extracellularly. Under hypoxic conditions, STAT3 can regulate the Rab7 and Rab27a proteins to induce EV secretion in ovarian cancer. These EVs increase cisplatin efflux in ovarian cells and might be responsible for chemoresistance [197].

### Hypoxic EVs mediate radioresistance

In cells, radiation is a genotoxic factor that can disrupt DNA and induce the release of ROS to inhibit cell proliferation. In BC management, radiotherapy acts as a crucial component [198] (Fig. 4B). Hypoxia influences the development of tumor radioresistance via the secretion of EVs. MiR-340-5p, miR-152-3p, ANGPTL4, and HSP70 are EV contents associated with radiation resistance in tumors under hypoxia. The detailed mechanisms include the activation of the Kruppel-like Factor 10 and the 15/UV radiation resistance-associated gene axis, which causes GSH peroxidase 4-induced ferroptosis suppression as well as lipid peroxidation and promotes tumor migration and angiogenesis [199–203]. In addition, some EVs, whose relationship with hypoxia needs further clarification, are closely related to the development of BC radioresistance. In BC cells, as the X-ray dose increases, the expression of several genes related to EVs, including Alix, Rab27a, Rab27b, TSPA8, and CD63, increases. Consequently, BC cells upregulate the biogenesis and secretion of these EVs, which help BC develop resistance to radiotherapy by promoting the apoptosis of NK cells [204, 205]. EVs secreted by radioresistant BC cells can also increase cell viability, colony formation, and the tumorsphere-forming ability in naive recipient cells to disseminate the resistant phenotype throughout the tumor [206]. Radiotherapy is widely used in the clinical treatment of BC, but the relevant mechanisms by which hypoxia affects the formation and transmission of radioresistance through EVs in BC remain unclear.

### Hypoxia and EVs mediate endocrine therapy resistance

Endocrine therapy targeting the estrogen receptor (ER) or progesterone receptor (PR) is a classic systematic treatment for hormone receptor + BC patients. Tamoxifen and Fulvestrant are typical endocrine drugs [207]. Hypoxia is a contributing factor to the development of endocrine resistance. First, it can alter ER-α to cause the drugs to lose their targets. In the regulatory intersection between hypoxia and estrogenic signaling in BC, HIF-α activation is responsible for the downregulation of ER-α, and a switch in the regulation of sodium-coupled neutral amino acid transporter 2 is the key link [105, 208]. Second, the development of endocrine resistance is associated with several hypoxic metabolic characteristics. LDH-B is expressed in endocrine-resistant BC cells, which increases lactic acid concentrations under hypoxia. The functions of increased lactate include enhancing BC cell motility and the phosphorylation level of extracellular regulated protein kinase 1/2 (ERK1/2), endowing Tamoxifen resistance to BC cells via EGFR/PI3K/Akt signaling, reducing intracellular E-cadherin expression, and inducing M2-like TAM polarization via the HIF-1α/STAT3 pathway [209, 210]. Third, hypoxia can cause endocrine resistance by increasing breast CSC activity. Mediators include the Notch, HIF, and integrin/Akt signaling cascades [211]. The role of EVs in the development of BC endocrine resistance should not be ignored. EVs containing metastasis-associated protein 1 from BC cells can facilitate ER signaling alterations and hypoxia in the TME, which results in BC resistance to hormone therapy [212]. In addition, EVs with miR-221, miR222, lncRNA UCA1, and mitochondrial RNA can spread endocrine resistance between BC cells via mechanisms such as decreasing ER-α expression, increasing CSC levels, increasing cell viability, and awaking dormant therapy-induced cancer stem-like cells, but their relationship with hypoxia needs further elucidation [207, 213, 214] (Fig. 4C).

### Hypoxia and EVs mediate resistance to targeted therapy

The HER2 and EGFR pathways are common targets of BC-targeted therapy, and representative drugs of these targets are Trastuzumab and Lapatinib, respectively [215]. Targeted gene mutation and downstream pathway activation are the resistance mechanisms most obviously affected by hypoxia and EVs [188]. For the drugs mentioned above, hypoxia is related to the development of resistance. For Trastuzumab, the main mediator of resistance development is HIF. After Trastuzumab therapy, HER2 + BC cells that overexpress HIF-1α become the major increased subpopulation, which indicates that cells with higher levels of HIF-1α could survive better under Trastuzumab by upregulating CD73 and CXCR4 expression levels [216–218]. Moreover, hypoxia can affect the sensitivity of BC to EGFR inhibitors such as Lapatinib. By regulating HIF-1 levels, hypoxia affects HIF-1 DNA binding activity and restores ERK pathway activity, resulting in the development of resistance to EGFR inhibitors in BC cells [219, 220]. EVs also play a role in the development of resistance to targeted drugs (Table 2). At present, several mechanisms of Trastuzumab resistance are relatively clear. BC cells secrete EVs with miR-146a-5p and neuromedin U, which target mechanisms such as increasing tumor migration and angiogenesis, reducing cyclin-dependent kinase N1A (CDKN1A) expression levels to promote cell cycle progression, and upregulating TGF-β1 and PD-L1 levels to spread Trastuzumab resistance [221, 222]. Apart from the EVs mentioned above, there are also EVs that transmit resistance through unelucidated mechanisms. The overexpression of thymidine kinase 1 (TK1) and CDK9 mRNAs in plasma-derived EVs is associated with Palbociclib resistance, and low expression of miR-630 in EVs is associated with Lapatinib resistance [223, 224]. Moreover, increased levels of miR-1246, miR-156, miR-21, miR-221, and lncRNA AGAP2-AS1 as well as proteins like pro-apoptotic protein PERP, GNAS2, GNA13, ITB1, and RAB10, and decreased levels of miR-375 in EVs can spread or enhance Trastuzumab resistance in BC [224–227]. However, detailed targets of these signaling molecules in EVs have not yet been discovered.

**Table 2:** EV contents associated with targeted therapy resistance

Molecules in EVs	Effect	Mechanism	Refer-ence
MiR-146a-5p↑	Spread Trastuzumab resistance	Promote cell cycle progression by reducing CDKN1A expression	[221]
NmU↑	Increase Trastuzumab resistance	Increase amounts of TGF-β1 and PD-L1 to inhibit anti-tumor immunity	[222]
TK1 and CDK9 mRNA↑	Increase Palbociclib resistance	Unknown	[223]
MiR-630 ↓	Increase Lapatinib resistance	Unknown	[224]
MiR-1246, miR-156, miR-21, miR-221, and lncRNA AGAP2-AS1↑	Spread or enhance Trastuzumab resistance of BC	Unknown	[225, 226]
Pro-apoptotic protein PERP, GNAS2, GNA13, ITB1, and RAB10↑	Spread or enhance Trastuzumab resistance of BC	Unknown	[227]
MiR-375 ↓	Spread or enhance Trastuzumab resistance of BC	Unknown	[227]

CDKN1A: cyclin-dependent kinase N1A; NmU: neuromedin U; TGF-β1: transforming growth factor-β1; PD-L1: programmed cell death ligand 1; TK1: thymidine kinase 1. ↑: Upregulation; ↓: Downregulation

At present, the development of drug resistance is a major obstacle to eradicating BC. In this section, we summarized the mechanisms by which BC cells develop resistance to different therapeutic methods. Considering that targeted therapy and endocrine therapy are relatively specific therapies for BC, future research focusing on the development of resistance to these two therapies may achieve greater benefits in overcoming this disease.

## Future directions of engineered EVs in treating BC

Engineered EVs are a technology that involves artificial modification of the surface molecules and contents of EVs to achieve targeted delivery of drugs to specific tissues or cells [228]. Owing to their low immunogenicity, lack of toxicity, and high cellular compatibility and targeting ability, engineered EVs are considered to enhance the efficiency, specificity, and safety of BC treatments by precisely delivering therapeutic agents [229]. Currently, EVs from various sources have been employed in research and clinical applications via diverse engineering techniques. This section provides a summary of the preparation methods of engineered EVs and their prospective application directions in the treatment of BC under hypoxic conditions.

### Engineered EV preparation methods

**Fig. 5:**
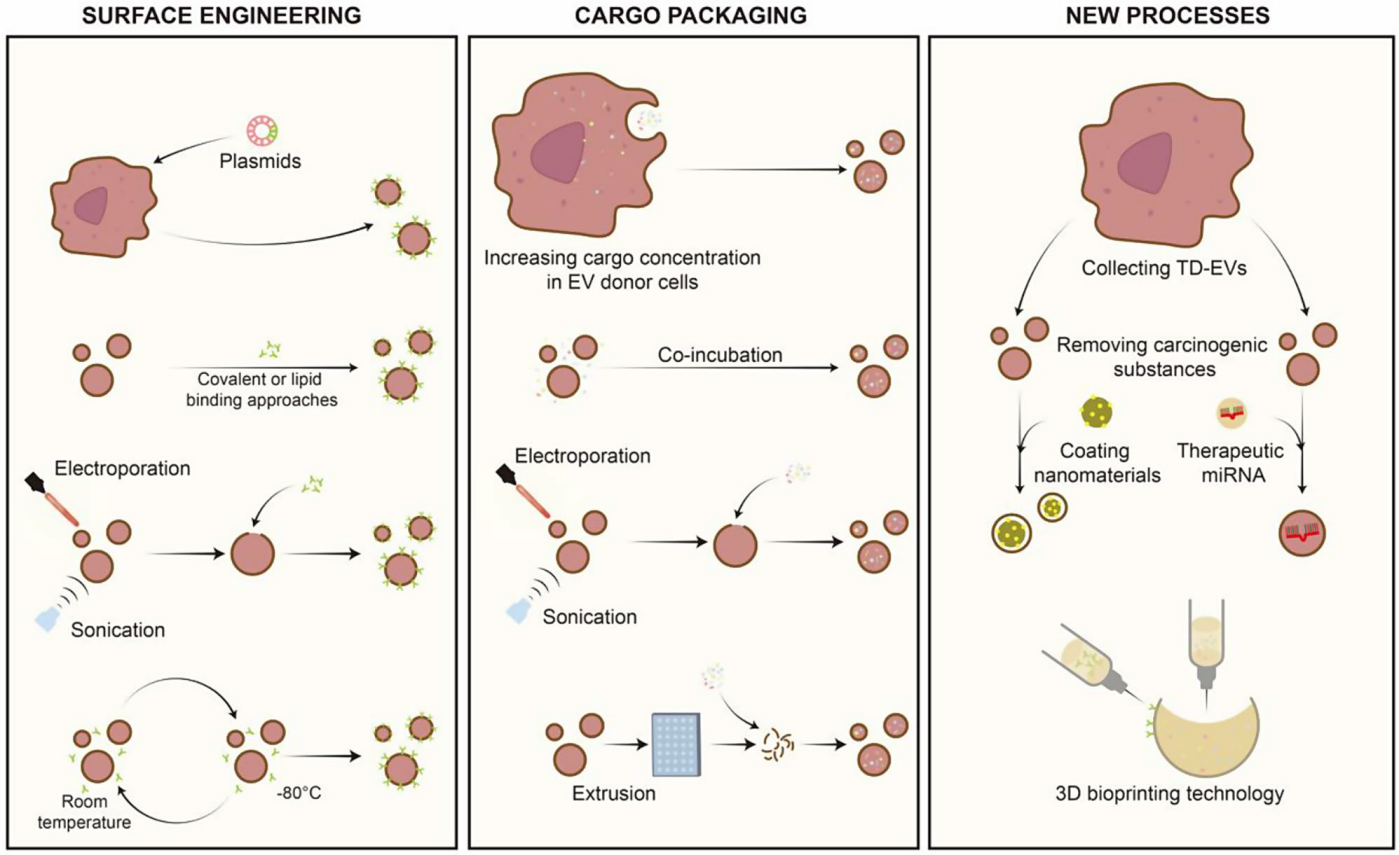
The preparation of engineered EVs. Specific surface engineering techniques, including genetic engineering, chemical conjugation, and physical methods, aim to obtain EVs with specific surface markers. Cargo packaging methods involve increasing the in situ loading of drugs within donor cells and various physical methods, such as coincubation and electroporation, to encapsulate specific drugs or molecules into EVs. Targeted high-yield engineered EVs can also be prepared via TD-EVs and 3D bioprinting technology

The technical aspects of the preparation of engineered EVs include surface engineering, which involves altering EV surface molecules for targeting and cargo packaging, which involves the encapsulation of specific drugs within EVs [230] (Fig. 5). Surface engineering methods include genetic, chemical, and physical modifications. Genetic modification involves modifying genes in EV-releasing cells to produce EVs with specific peptide chains or ligands with plasmids as the commonly used vectors [231]. Chemical modification operates at the protein level, modifying ligands on the EV surface, and it primarily involves covalent or lipid binding approaches to attach specific recognition molecules to the EV surface [230, 232]. The physical methods used include electroporation, ultrasound, and freeze-thaw techniques [232]. When engineered EVs target hypoxic BC cells, to make engineered EVs more efficient toward the hypoxic BC TME, it is necessary to use the above methods so that the EV surface can carry recognition molecules that can target BC cells while also improving the ability of EVs to respond to hypoxia. The surface molecules that can guide the binding of engineered EVs to BC cells include anti-human HER2 antibodies, mesenchymal-epithelial transition factor-binding peptide, aptamer AS1411, and lipidomimetic chain-conjugated HA [233–236]. The currently constructed hypoxia-responsive drug delivery systems are mainly based on liposomes and polymer nanoparticles. Nitroimidazole, azobenzene fragments, and metal (such as cobalt) complexes are the three most commonly used surface structures that can endow the delivery system with hypoxia responsiveness [237–239]. Reports on the construction of hypoxia-responsive drug delivery systems using EVs as carriers are lacking at present, possibly because EVs have more complex components and structures than liposomes and polymer particles do, making the production of EVs with the aforementioned surface structures more difficult [240]. However, owing to the outstanding advantages of EVs in terms of cell compatibility, biological toxicity, and other aspects, the construction of hypoxia-responsive engineered EVs is worthy of further exploration.

The means used to achieve cargo packaging can be divided into two categories: increasing the cargo concentration within EV donor cells by enhancing in situ cargo preloading of EVs and using methods such as coincubation, electroporation, sonication, and extrusion to introduce cargo into free EVs [230, 241]. Engineered EVs can carry a diverse range of cargo, including nucleic acid fragments, proteins, lipids, metabolites, and various drugs, with the expectation that they can exert therapeutic effects in tumor tissues [242]. Maryam et al. reported that by incorporating appropriate cleavage sites, cargo sorting can occur within the lumen of EVs, which further expands the applications of engineered EVs [243]. The specific drugs or molecules that can be encapsulated in engineered EVs and delivered to hypoxic BC tissues through the aforementioned techniques to alleviate hypoxia in the BC TME or reduce the malignant phenotypes caused by hypoxia in BC are described in detail in the next section.

In addition to traditional processes, the preparation of engineered EVs can be achieved through 3D bioprinting technology. This approach offers advantages such as personalization and tunable degradation for sustained delivery, but its primary applications are currently in tissue engineering, with its value in cancer treatment awaiting further exploration [244]. In addition, TD-EVs also offer an alternative approach to preparing engineered EVs. By collecting TD-EVs and employing methods such as electroporation to remove carcinogenic substances, highly targeted TME-derived EVs can be obtained. Loading drugs into such EVs and applying these EVs as coatings on nanocarriers also results in excellent tumor tissue specificity, and this approach has been explored for BC treatment [245, 246]. In addition, under hypoxia, EVs produced by MSCs scavenge ROS, induce M2 macrophage polarization, and stabilize HIF-1α in target cells, whereas EVs produced by ADSCs and umbilical cord-derived MSCs promote EC survival and angiogenesis, which may contribute to tumor progression [247–249]. These findings suggest that treating donor cells with hypoxia can alter the effects of engineered EVs.

### Prospects for engineered EV therapy for BC

The use of engineered EVs for BC treatment involves another crucial aspect: selecting appropriate drugs or molecules for encapsulation within EVs to ameliorate the hypoxic conditions in the BC TME or mitigate the malignant phenotypes in hypoxic settings [250]. In this section, we present certain drugs or molecules with the hope that future research will explore their combination with engineered EV technology. This integration might enable precise delivery to tumor tissues, effectively addressing the malignant phenotypes of hypoxic BC, including invasive and metastatic properties, angiogenesis, abnormal metabolism, immune dysregulation, and drug resistance (Table 3).

First, specific drugs or molecules have demonstrated therapeutic efficacy in addressing the increased invasiveness and metastatic potential of BC in hypoxic environments. MiR-140-5p, which targets Nrf2, regulates the Nrf2/HO-1 axis to inhibit BC progression [251]. In addition, the following drugs discussed can inhibit the invasiveness of tumors by inhibiting HIF-1α and its downstream reactions. DHA, ION-31a, and Genistein effectively downregulate VEGF expression in tumor cells by suppressing the HIF-1α pathway, thereby inhibiting BC proliferation and metastasis [252–254]. The traditional Chinese medicine Sanguisorba officinalis L. can inhibit HIF-1α/Caveolin-1 signaling, BC growth, and EMT [255]. Additionally, Bishonokiol A reduces HIF-1α synthesis and MMP-9 activity to decrease cell invasion and migration [256]. Considering the poor efficacy of conventional chemotherapy drugs on tumor metastasis, the combination of engineered EVs with these drugs may be a good supplement to the existing treatment options for controlling BC metastasis.

Second, when increased angiogenesis in BC tissues under hypoxia is addressed, specific drugs exhibit targeted effects. Considering that TD-EVs promote tumor angiogenesis in many ways, targeting angiogenesis with engineered EVs is hypothesized to achieve good therapeutic effects [257]. Melatonin (MLT) not only has an endocrine function but also downregulates the HIF-1/ROS/VEGF axis, inhibiting human umbilical vein EC (HUVEC) activity and angiogenesis [258]. In addition, Simvastatin can inhibit angiogenesis by phosphorylating AMPK and downregulating HIF-1 transcription [259].

Third, under hypoxic conditions, BC cells exhibit pronounced metabolic abnormalities, such as the Warburg effect and changes in lipid metabolism, which promote tumor growth. MLT, a flavonoid, honokiol, and coenzyme Q(0) extracted from Antrodia camphorata can downregulate HIF-1α expression, inhibiting the Warburg effect to reduce tumor cell glycolysis [260–263]. Moreover, a compound named LW1564 inhibits mitochondrial metabolism. Future attempts could utilize engineered EVs to transport LW1564 into BC mitochondria because they can inhibit mitochondrial oxygen consumption, increase intracellular oxygen concentrations, stimulate HIF-1α degradation, and correct abnormal metabolism in tumor cells [264]. In lipid metabolism, the HIF-1α inhibitor YC-1, in combination with palmitic acid and L-carnitine, activates fatty acid β-oxidation and induces ROS overproduction, promoting liver cancer cell apoptosis [265]. Exploring whether similar mechanisms exist in BC is worthwhile.

Fourth, certain drugs enhance the dormant antitumor immune properties of the BC TME under hypoxia. Pantothenate and its metabolite CoA reprogram T lymphocytes to polarize them toward antitumor immunity by regulating HIF-1α and the aryl hydrocarbon receptor [266]. Zoledronic acid (ZA) reverses BC resistance to immunotherapy. Delivering ZA to tumor tissues via nanoparticles reduces Ras/ERK1/2/HIF-1a axis activity and P-glycoprotein expression and, as a result, increases the number of DCs and reduces the number of infiltrating Tregs [267]. Further attempts could use engineered EVs with higher targeting specificity instead of liposomes, aiming for better efficacy. Studies have shown that transporting hemoglobin to the TME via liposomes alleviates local hypoxia and enhances the efficacy of PD-1 antibodies against BC [268]. Similarly, in the future, engineered EVs can be used to replace liposomes to observe whether better efficacy can be achieved. Additionally, the use of EVs to deliver small interfering RNAs (siRNAs) targeting HIF-1α and STAT3 to BC tissues might increase CTL and IFN-γ levels, thereby enhancing local antitumor immunity in the BC TME [269].

Finally, the different types of therapeutic resistance developed by BC under hypoxia can potentially be addressed by certain drugs. Metformin enhances BC sensitivity by targeting various pathways, such as the mTOR, ERK/P70S6K, NF-κB/HIF-1α, and mitogen-activated protein kinase (MAPK) pathways [270]. In the context of radioresistance, wogonin, a primary component of Scutellaria baicalensis, can increase the expression of Keap1, an Nrf2 inhibitor, to inhibit the Nrf2/HIF-1α pathway to promote BC cell apoptosis under radiation [271]. Moreover, para-toluenesulfonamide upregulates apoptosis-related protein expression and downregulates CA9, HIF-1α, and VEGF protein expression, potentially preventing BC patients from developing resistance to α-PD-1 therapy [272]. For targeted therapy resistance caused by increased drug efflux, the monoacylglycerol lipase inhibitor JJKK048 improves resistance by increasing the intracellular concentration of Regorafenib in TNBC [273].

In summary, this section outlined traditional and cutting-edge approaches for preparing engineered EVs and lists drugs aimed at inhibiting HIF-related pathways or improving the hypoxic state of BC cells to alleviate the malignant phenotype of tumors. It is hoped that in the future, these drugs can be delivered via engineered EVs to achieve better efficacy. However, the widespread clinical application of engineered EVs currently faces significant challenges, including difficulties in preparation and low yields. To overcome these obstacles, future efforts should focus on improving the efficiency of cargo loading into EVs, refining EV extraction methods, and enhancing the yield of EVs from donor cells.

**Table 3:** Drugs and molecules that can reduce the malignant phenotype of BC under hypoxia

Drug/Molecule	Target Molecule/Pathway	Effect	Reference
MiR-140-5p	Nrf2/HO-1 axis	Inhibit BC progression	[251]
DHA, ION-31a and Genistein	HIF-1α/VEGF pathway	Inhibit BC proliferation and metastasis	[252–254]
Sanguisorba officinalis L.	HIF-1α/Cav-1 signaling	Inhibit BC growth and EMT	[255]
Bishonokiol A	HIF-1α and MMP-9	Decrease cell invasion and migration	[256]
MLT	HIF-1/ROS/VEGF axis	Inhibit HUVEC activity and angiogenesis	[258]
Simvastatin	AMPK and HIF-1	Inhibit angiogenesis	[259]
MLT, Flavonoids and Honokiol	HIF-1α	Inhibit Warburg effect and reduce tumor cell glycolysis	[260–262]
Co Q(0)	HIF-1α	Suppress TNBC’s Warburg effect, EMT, and inflammatory response	[263]
LW1564	Mitochondrial metabolism and HIF-1α	Correct tumor cell abnormal metabolism	[264]
YC-1	FAO	Induce ROS overproduction and promote liver cancer cell apoptosis	[265]
Pantothenate and CoA	HIF-1α and AhR	Reprogram T lymphocytes to polarize them toward anti-tumor immunity	[266]
ZA	Ras/ERK1/2/HIF-1a axis and P-glycoprotein	Increase DC and reduce infiltrating Tregs	[267]
Hemoglobin	—	Alleviate local hypoxia and enhance PD-1 efficacy	[268]
SiRNA of HIF-1α and STAT3	HIF-1α and STAT3	Increase CTL and IFN-γ levels	[269]
Metformin	mTOR, ERK/P70S6K, NF-κB/HIF-1α, and MAPK	Enhancing chemotherapy sensitivity	[270]
Wogonin	Nrf2/HIF-1α pathway	Promote BC cell apoptosis and reduce BC radiotherapy resistance	[271]
PTS	Apoptosis-related protein, CA IX, HIF-1 α, and VEGF	Prevent potential resistance to α PD-1 therapy	[272]
JJKK048	MAGL	Increase intracellular Regorafenib concentration in TNBC	[273]

DHA: Docosahexaenoic acid; HIF: Hypoxia-induced factor; VEGF: Vascular endothelial growth factor; Cav: Caveolin; EMT: Epithelial-to-mesenchymal transition; MMP-9: Matrix metalloprotease-9; MLT: Melatonin; ROS: Reactive oxygen species; AMPK: Adenosine 5’-Monophosphate-activated protein kinase; HUVEC: Human umbilical vein endothelial cell; CoQ(0): Coenzyme Q(0); TNBC: Triple-negative BC; FAO: Fatty acid β-oxidation; AhR: Aryl hydrocarbon receptor; ZA: Zoledronic acid; DC: Dendritic cell; CTL: Cytotoxic lymphocyte; MAPK: Mitogen-activated protein kinase; MAGL: Monoacylglycerol lipase; PTS: Para-toluenesulfonamide; CA IX: Carbonic anhydrase 9

## Conclusion and future prospects

Hypoxia is a significant characteristic of solid tumors. This leads to a low-oxygen and low-pH environment, impairing the function of antitumor immune cells and attracting various immunosuppressive cells to provide an immunosuppressive TME for tumor cells to survive. Furthermore, numerous studies have indicated that hypoxia-induced EV-mediated communication in the TME is related to many malignant phenotypes in BC. Owing to their targeting properties and protective effects on their contents, EVs have become the main means of long-distance intercellular communication.

In this review, we first summarized how EVs mediate communication between different components of the hypoxic TME. Next, we investigated the roles that EVs play in altering tumor cell metabolic characteristics and metabolic reprogramming events in the hypoxic TME. As tumors continue to progress, the scarcity of nutrients forces tumor cells to solve malnutrition through metastasis, while EVs help tumor cells survive in metastatic foci and establish new tumor tissues by shaping the PMN of tumors. The TME and metabolic reprogramming mentioned above jointly shape the occurrence of therapeutic resistance in BC. Therefore, the mechanisms by which EVs mediate BC therapeutic resistance under hypoxia have also been elucidated. Deciphering the above key mechanisms can better guide clinical treatments. Finally, owing to the high biocompatibility, low immunogenicity, high drug delivery efficiency, and ease of modification of EVs, we summarized the future directions of engineered EVs for the treatment of the aforementioned malignant phenotypes and potential therapeutic targets.

In summary, this review primarily focuses on the formation, metabolic reprogramming, metastasis, and drug resistance of EVs in the hypoxic TME of BC. This understanding can help us better comprehend the biological processes of BC and thus address this clinical challenge more effectively.

## Data Availability

No datasets were generated or analysed during the current study.

## Abbreviations

BC: Breast cancer
TME: Tumor microenvironment
HIF: Hypoxia-inducible factor
EMT: Epithelial-to-mesenchymal transition
EV: Extracellular vesicle
ECM: Extracellular matrix
TNBC: Triple-negative breast cancer
MMP: Matrix metalloprotease
IFP: Interstitial fluid pressure
PD-L1: Programmed cell death ligand 1
TAM: Tumor-associated macrophage
Treg: Regulatory T lymphocyte
TD-EV: Tumor-derived extracelluar vesicle
CSF: Colony-stimulating factor
TGF-β: Transforming growth factor-β
EC: Endothelial cell
lncRNA: Long noncoding RNA
CXCL: Chemokine C-X-C motif ligand
NK cell: Natural killer cell
MC: Mesenchymal cell
OXPHOS: Oxidative phosphorylation
DC: Dendritic cell
CCR: C-C chemokine receptor
IL: Interleukin
VEGF: Vascular endothelial growth factor
PMN: Premetastatic niche
TAN: Tumor-associated neutrophil
ROS: Reactive oxygen species
NET: Neutrophil extracellular trap
CTC: Circulating tumor cell
HSP: Heat shock protein
CTL: Cytotoxic lymphocyte
CAF: Cancer-associated fibroblast
CAF-EV: Cancer-associated fibroblast-derived extracellular vesicle
CSC: Cancer stem cell
PKM: Pyruvate kinase M
LDH: Lactate dehydrogenase
CA IX: Carbonic anhydrase 9
SREBP-1: Sterol regulatory element-binding protein-1
Gln: Glutamine
GLUT: Glucose transporter
FAS: Fatty acid synthesis
FATP: Fatty acid transport protein
FABP: Fatty acid binding protein
FASN: Fatty acid synthetase
27-HC: 27-hydroxycholesterol
MiRNA/miR: Micro RNA
GSH: Glutathione
PPP: Pentose phosphate pathway
Nrf2: Nuclear factor erythroid 2-related Factor 2
AMPK: Adenosine 5’-monophosphate-activated protein kinase
ZEB: Zinc-finger E-box binding
LOX: Lysyl oxidase
FGF: Fibroblast growth factor
ADSC: Adipose-derived mesenchymal stem cell
HUVEC: Human umbilical vein endothelial cell
G-CSF: Granulocyte colony-stimulating factor
ANGPTL4: Angiopoietin-like 4
STAT: Signal transducer/activator of transcription
CircRNA: Circular RNA
ER: Estrogen receptor
PR: Progesterone receptor
CDK: Cyclin-dependent kinase
TK1: Thymidine kinase 1
SiRNA: Small interfering RNA
ERK: Extracellular regulated protein kinase
MLT: Melatonin
ZA: Zoledronic acid
MAPK: Mitogen-activated protein kinase
MSC: Mesenchymal stem cell
